# Updated list of Tersilochinae (Hymenoptera, Ichneumonidae) of Italy

**DOI:** 10.3897/BDJ.12.e139683

**Published:** 2025-03-07

**Authors:** Filippo Di Giovanni, Davide Dal Pos, Andrey I. I. Khalaim

**Affiliations:** 1 University of Siena, Siena, Italy University of Siena Siena Italy; 2 University of Central Florida, Orlando, United States of America University of Central Florida Orlando United States of America; 3 Universidad Autónoma de Tamaulipas, Cd. Victoria, Mexico Universidad Autónoma de Tamaulipas Cd. Victoria Mexico; 4 Russian Academy of Sciences, St. Petersburg, Russia Russian Academy of Sciences St. Petersburg Russia

**Keywords:** *Aneuclis*, biological control, checklist, Darwin wasps, diversity, parasitoids

## Abstract

**Background:**

The subfamily Tersilochinae is a small taxon that accounts for about 60 species in Italy. However, the current checklist of the group is incomplete and listed records are often imprecise.

**New information:**

An updated checklist of the Italian Tersilochinae is provided. Three species are new additions to the Italian fauna, while three others are first records for southern Italy. Additionally, *Aneuclispusilla* Masi, 1933 is reported as a junior synonym of *Aneuclismelanaria* (Holmgren, 1860) **syn. nov**. This update raises the total number of Tersilochinae species in Italy to 77.

## Introduction

Tersilochinae is a relatively small subfamily of Darwin wasps (Hymenoptera, Ichneumonidae) comprising around 600 species worldwide, except for Antarctica (Yu et al. 2016, Khalaim 2022). Following the phylogenetic analysis of Quicke et al. (2009), the subfamily was expanded to include members of the subfamily Neorhacodinae and the “microphrudine” genera of the former subfamily Phrudinae (whereas the other Phrudinae were placed in the subfamily Sisyrostolinae). Subsequently, Broad et al. (2018) reinstated Neorhacodinae as a separate subfamily, thus leading to the current definition of the subfamily Tersilochinae (Broad et al. 2018, Bennett et al. 2019). The group comprises small to medium-sized species, most of which act as solitary koinobiont endoparasitoids of Coleoptera larvae (with some exceptions in the genera *Gelanes* Horstmann and *Tersilochus* Holmgren) (Broad et al. 2018).

In Italy, a checklist of all Ichneumonidae dates back to 1995 and reports about 1,850 species, of which about 60 belong to the subfamily Tersilochinae (Scaramozzino 1995). Subsequent studies increased the number of Darwin wasp species known for the country to more than 2,260 (Di Giovanni et al. 2015, Yu et al. 2016, Di Giovanni and Reshchikov 2016, Kostro-Ambroziak and Di Giovanni 2016, Di Giovanni and Riedel 2017, Di Giovanni and Scaramozzino 2019, Korenko and Di Giovanni 2019). However, the actual number of ichneumonids in Italy is likely much higher (Di Giovanni et al. 2015). Indeed, although the 1995 checklist was an ambitious project, which laid the foundations for the current faunal surveys in the Italian territory (Minelli et al. 1993-1995), the necessity to meet the project’s established deadline resulted in a reduction of information (Minelli 1996). Furthermore, the limited distribution of the work and its publication in Italian led Yu et al. (2016) to overlook Scaramozzino (1995)'s checklist, resulting in inconsistencies in the reported number of Ichneumonidae species in these two works (Di Giovanni et al. 2015).

In Ichneumonidae, the lack of precise references to the consulted literature and voucher specimens in collections further complicates record verification and tracking of taxonomic and systematic changes that have occurred since the publication of Scaramozzino (1995)'s checklist. Furthermore, the geographical division adopted in the checklist, that basically divides the Italian peninsula into two macro-areas (northern and southern Italy) plus the two largest islands, Sicily and Sardinia, provides an approximate and fragmentary picture of the distribution of the known species in Italy.

Below, an updated list of the Italian Tersilochinae is reported. Through a literature review and new records, distribution data for the Italian species of the group are reported, where possible, based on the 20 administrative regions into which the Italian territory is divided. Three species are new records for the Italian fauna and three species are reported for the first time in southern Italy. Additionally, the status of *Aneuclispusilla* Masi, 1933 is clarified, synonymising it with *A.melanaria* (Holmgren, 1860). The present study represents a small step towards filling the gaps in the knowledge of Darwin wasp fauna of Italy, contributing to the development of a more comprehensive checklist of Italian fauna.

## Materials and methods

The previous checklist of Italian fauna (Minelli et al. 1993-1995) reported information on species subdividing Italian territory into four geographical areas: North Italy, including Friuli Venezia Giulia (FVG), Veneto (VEN), Trentino-Alto Adige (TAA), Lombardy (LOM), Val d’Aosta (VAO), Piedmont (PIE), Liguria (LIG) and Emilia-Romagna (EMR); South Italy, including Tuscany (TOS), Marche (MAR), Umbria (UMB), Latium (LAZ), Abruzzo (ABR), Molise (MOL), Campania (CAM), Apulia (PUG), Basilicata (BAS) and Calabria (CAL); Sicily (SIC); and Sardinia (SAR). In the present work, species have been reported, where possible, according to the division of Italy into administrative regions. Where the region cannot be traced back, the previous division into North and South Italy or the generic indication "Italy" is used. Misspellings are indicated with [sic] following the incorrect generic or specific epithet. It should be noted that misattributions (i.e. an error in the authorship of a name) are common in ancient literature; we avoided reporting them, except where they contribute to possible confusion between different species.

Since it is beyond the scope of the current contribution, we did not corroborate earlier records by examining the actual specimens reported by previous authors.

### Photographs

Pictures of three species newly recorded from Italy were taken using an Olympus OM-D E-M1 digital camera attached to an Olympus SZX10 stereomicroscope and partially focused images were combined using Helicon Focus Pro (ver. 7.6.6) software. Images of the type of *Aneuclispusilla* Masi, 1933 were taken using a Zeiss Axio Zoom V16 microscope and stacked using Zerene Stacker software ver. 1.04.

### List of depositories

- **FDGC** = Filippo Di Giovanni private collection, Siena, Italy
- **MSNGD** = Museo di Storia Naturale Giacomo Doria, Genoa, Italy
- **NHMUK** = The Natural History Museum, London, UK
- **RMNH** = Rijksmuseum van Natuurlijke Historie, Leiden, Netherlands
- **ZISP** = Zoological Institute of Russian Academy of Sciences, St Petersburg, Russia

### Treatment of taxa

Morphological terminology follows Broad et al. (2018). For wing veins, the terminology used by Townes (1969) is also given. Species general distribution follows Yu et al. (2016).

## Checklists

### Checklist of Italian Tersilochinae

#### Allophroides

Horstmann, 1971

E585A5D1-CA6B-57EB-A2F3-695F9B7B7C6E

#### Allophroides boops

(Gravenhorst, 1829)

6EEC36A4-C2DA-57AD-9AE9-7E3C50D82415

##### Distribution

Palaearctic (Western Palaearctic).

**Asserted distribution in Italy**: Italy (Horstmann 1971); North Italy (Scaramozzino 1995); PIE (Gravenhorst 1829, as *Porizonboops* Gravenhorst, 1829; Frilli and Horstmann 1982, as *Ophionitalicus* (Gravenhorst, 1829)); LIG (Gravenhorst 1829, as *Porizonitalicus* Gravenhorst, 1829); EMR (Zangheri 1969, as *Allophrysboops* (Gravenhorst, 1829)).

##### Notes

Horstmann (1971) recorded the species for "? Italy (Gravenhorst)", acknowledging uncertainty in the synonymisation of the sole damaged type specimen of *Porizonitalicus* Gravenhorst, 1829 in Gravenhorst’s collection with this species.

#### Aneuclis

Förster, 1869

8B562B5B-D123-5C5A-A557-5441B033A9F9

#### Aneuclis incidens

(Thomson, 1889)

E7D07473-A60D-592F-970F-BEF626935F40

##### Materials

- **Type status:** Other material. **Occurrence:** recordedBy: J. Osborne; individualCount: 1; sex: female; occurrenceID: 4F846C11-4462-534F-BD0E-C7982EDE365D; **Location:** country: Italy; countryCode: IT; stateProvince: Campania [CAM]; locality: near Naples; **Identification:** identifiedBy: A. Khalaim; **Event:** year: 1966; month: 8; day: 22; verbatimEventDate: 22.viii.1966; **Record Level:** institutionCode: NHMUK

##### Distribution

Palaearctic (Eastern Palaearctic; Western Palaearctic).

**Asserted distribution in Italy**: North Italy (Scaramozzino 1995); TAA (Horstmann 1981); South Italy (Scaramozzino 1995, Horstmann 1981); CAM (**new record**).

#### Aneuclis maritima

(Thomson, 1889)

643E514F-4062-5F0B-9512-49D5B34D03DA

##### Distribution

Palaearctic (Eastern Palaearctic; Western Palaearctic).

**Asserted distribution in Italy**: EMR (Zangheri 1969).

#### Aneuclis melanaria

(Holmgren, 1860)

FBE7D15F-B22E-5418-A7D4-F44240A85B38

= *Aneuclispusilla* Masi, 1933. Holotype (♀): Italy, Tuscany, Capraia. **New synonym.**

##### Materials

- **Type status:** Holotype. **Occurrence:** individualCount: 1; sex: female; occurrenceID: 9611EE52-C103-5305-9F37-9736FC2E18F7; **Location:** country: Italy; countryCode: IT; stateProvince: Tuscany [TOS]; locality: Capraia, Porto; verbatimLocality: CAPRAIA Tosc. / Porto; **Event:** year: 1931; month: 9; verbatimEventDate: IX ∙ 1931; **Record Level:** institutionCode: MSNGD

##### Distribution

Palaearctic (Eastern Palaearctic; Western Palaearctic).

**Asserted distribution in Italy**: North Italy (Scaramozzino 1995); PIE (Pagliano 2009); EMR (Zangheri 1969); South Italy (Scaramozzino 1995, also as *Aneuclispusilla* Masi, 1933); TOS (Masi 1933, as *Aneuclispusilla* Masi, 1933); LAZ (Pesarini 2009); ABR (Pagliano 2009); SIC (Horstmann 1981, Scaramozzino 1995).

##### Notes

**Original type label for *Aneuclispusilla* Masi, 1933**

Typus // CAPRAIA Tosc. / IX ∙ 1931 Porto / C. Mancini // Aneuclis / pusillus / Typus! ♀ Ms. Museo Civico / di Genova

**Description of the type of *Aneuclispusilla* Masi, 1933**

**Figure 1.**
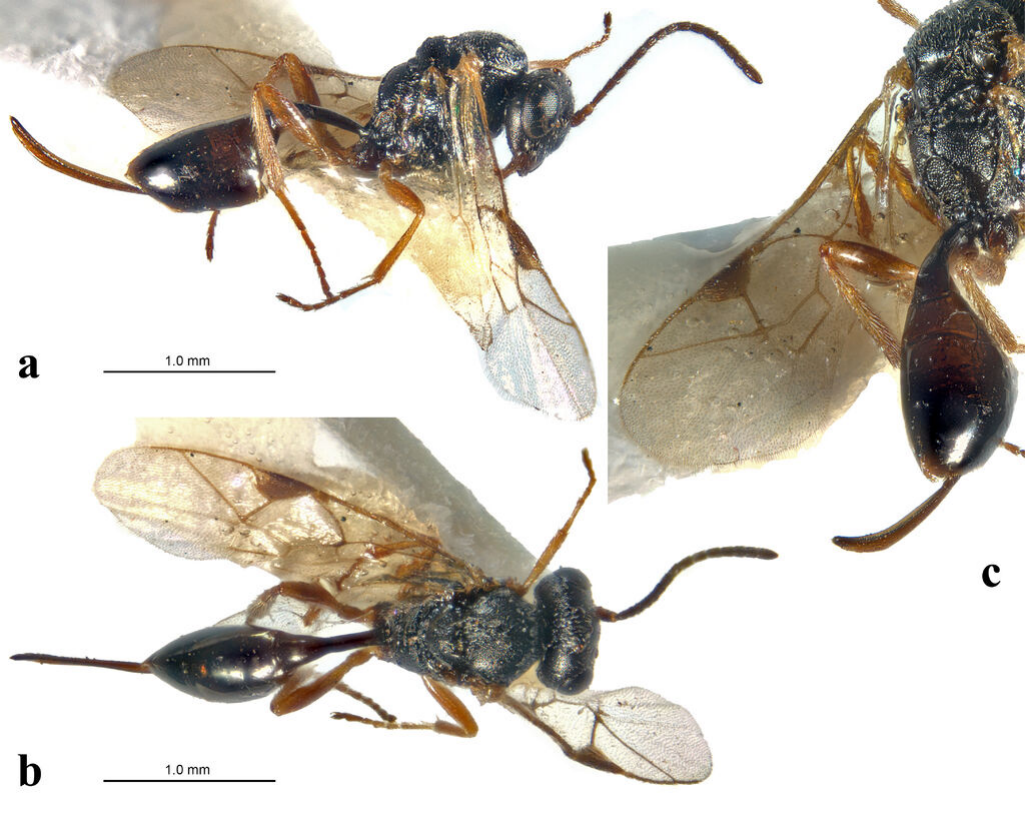
*Aneuclispusilla* Masi, 1933, syn. *Aneuclismelanaria* (Holmgren, 1860), syn. nov.; **a-c** female, holotype (MSNGD); **a** habitus, lateral view; **b** habitus, dorsal view; **c** propodeum and metasoma, dorso-lateral view.

Female (Fig. 1). Body length 2.6 mm (without ovipositor); ovipositor 0.8 mm. Fore wing length 2.2 mm.

Head, in dorsal view, gently constricted posterior to eyes, about 0.6× as long as eye width. Clypeus about 2.5× as broad as long, lenticular in frontal view, coriaceous on basal 1/3, polished and without punctures on lower 1/3. Mandible approximately 3.0× as long as basally wide, teeth subequal. Malar space about 1.1× as long as basal mandibular width, coriaceous. Antennal flagellum with 15 flagellomeres. Face, frons, vertex and gena coriaceous and sub-polished.

Fore-wing with vein 2m-cu postfurcal (i.e. second recurrent vein), with bulla occupying more than its upper half; vein 2rs-m obliterated (i.e. first intercubitus); 2r&RS straight (i.e. proximal section of radius), about 1.25× as long as the width of the pterostigma, meeting RS (i.e. distal section of radius) at about 85°; pterostigma broad, subtriangular; RA (i.e. metacarpus) not reaching apex of fore-wing; vein 1cu-a (i.e. nervulus) clearly distal to M&RS (i.e. basal vein) for a distance almost equal to its length; vein 2cu-a (i.e. postnervulus) almost obliterated. Hind-wing with vein CU+cu-a (i.e., nervellus) straight, clearly reclivous.

Legs slender. Hind femur about 4.4× as long as maximum width. Hind tarsal claws not pectinate.

First metasomal tergite about 3.0× as long as posteriorly broad, smooth and polished, without punctures; glymma weak, elongated. Second metasomal tergite transverse, about 0.75× as long as posteriorly broad. Thyridial depression elongated, coriaceous. Ovipositor upcurved, sheath about 1.3× as long as hind tibia.

Black. Scapus, pedicel, antennal flagellum basally, clypeus, mandible (except black teeth), palps reddish-brown. Coxae black, legs red, tarsi reddish-brown. Pterostigma yellowish-brown. Sheath brownish-black.

**Further Remarks**

We have examined the type of *A.pusilla* and found that it is a typical *A.melanaria*. It should be noted that in the original description (Masi 1933), *A.pusilla* was compared with *Aneuclisbrevicauda* (Thomson, 1889) and *Problesexilis* (Holmgren, 1860), but not with *A.melanaria*.

#### Aneuclis pumilus

(Holmgren, 1860)

70C4316B-EC25-5C15-837E-50C62EF1D62B

##### Materials

- **Type status:** Other material. **Occurrence:** recordedBy: M. Bardiani; D. Birtele; individualCount: 2; sex: females; occurrenceID: F39AF1FE-6FED-54C0-9B93-0F01AF818C67; **Location:** country: Italy; countryCode: IT; stateProvince: Lombardy [LOM]; municipality: Mantova; locality: Marmirolo, Bosco della Fontana; verbatimElevation: 25 m; **Identification:** identifiedBy: A. Khalaim; **Event:** samplingProtocol: Malaise trap on canopy (15.5–17.0 m); year: 2008; month: 9; day: 2-30; verbatimEventDate: 02–30.ix.2008; **Record Level:** institutionCode: FDGC

##### Distribution

Afrotropical; Australasian; Indomalayan; Nearctic; Neotropical; Palaearctic (Eastern Palaearctic; Western Palaearctic).

**Asserted distribution in Italy**: North Italy (Scaramozzino 1995, as *Sathropteruspumilus* (Holmgren, 1860)); LOM (Khalaim 2004, as *Sathropteruspumilus* (Holmgren, 1860); **new record**); PIE (Pagliano 2009, as *Sathropteruspumilus* (Holmgren, 1860)); EMR (Zangheri 1969, as *Sathropteruspumilus* (Holmgren, 1860)).

#### Astrenis

Förster, 1869

99CB49FC-AFFE-548A-A3A2-47D8B611784D

#### Astrenis brunneofacies

Vikberg, 2000

93E47520-F273-59FF-BF1A-B01DABCC5827

##### Distribution

Palaearctic (Western Palaearctic).

**Asserted distribution in Italy**: VEN (Vikberg and Koponen 2000); TAA (Vikberg and Koponen 2000).

#### Barycnemis

Förster, 1869

DF50D62C-1BD0-53BA-9DEE-21AD97D7BE0B

#### Barycnemis alpina

(Strobl, 1901)

4C8DCA2A-A971-552D-9161-CFACBCCC9340

##### Distribution

Palaearctic (Eastern Palaearctic; Western Palaearctic).

**Asserted distribution in Italy**: North Italy (Horstmann 1981, Scaramozzino 1995); TAA (Khalaim 2016, Khalaim 2019).

#### Barycnemis angustipennis

(Holmgren, 1860)

01BB1F5B-1472-5C6C-AB46-13A78D453D6A

##### Distribution

Palaearctic (Eastern Palaearctic; Western Palaearctic).

**Asserted distribution in Italy**: North Italy (Horstmann 1981, Scaramozzino 1995); TAA (Smits van Burgst 1914, as *Cratophionangustipennis* (Holmgren, 1860); Smits van Burgst 1918, as *Cratophionangustipennis* (Holmgren, 1860)); VAO (Pagliano 2009); PIE (Pagliano 2009).

#### Barycnemis bellator

(Müller, 1776)

5A0545A1-27F3-5061-A00C-75F0D3AD1333

##### Distribution

Nearctic; Palaearctic (Eastern Palaearctic; Western Palaearctic).

**Asserted distribution in Italy**: North Italy (Scaramozzino 1995).

#### Barycnemis dissimilis

(Gravenhorst, 1829)

D77C1EE4-CA77-5541-9949-167B2A7F0F84

##### Distribution

Indomalayan; Nearctic; Palaearctic (Eastern Palaearctic; Western Palaearctic).

**Asserted distribution in Italy**: North Italy (Scaramozzino 1995); TAA (Khalaim 2016, Khalaim 2019); LOM (Khalaim 2016); PIE (Pagliano 2009); SIC (De Stefani 1895, as *Tersilochusdissimilis* (Gravenhorst, 1829)).

#### Barycnemis exhaustator

(Fabricius, 1798)

5BA9C562-1CF5-502B-A990-D16F4B7B82E6

##### Distribution

Palaearctic (Eastern Palaearctic; Western Palaearctic).

**Asserted distribution in Italy**: North Italy (Scaramozzino 1995, as *Barycnemisexhaustor* [sic] (Fabricius, 1798)); TAA (Horstmann 1981, as *Barycnemisexhaustor* [sic] (Fabricius, 1798)).

#### Barycnemis filicornis

(Thomson, 1889)

9C770E7F-D045-56AA-BAC2-763B2E27898D

##### Distribution

Palaearctic (Western Palaearctic).

**Asserted distribution in Italy**: North Italy (Horstmann 1981, Scaramozzino 1995); TAA (Khalaim 2016, Khalaim 2019); LOM (Khalaim 2016); PIE (Pagliano 2009); EMR (Zangheri 1969, as *Leptopygusfilicornis* (Thomson, 1889)).

#### Barycnemis gravipes

(Gravenhorst, 1829)

882B1514-CE82-5799-95CE-60B19C7907F2

##### Distribution

Nearctic; Palaearctic (Eastern Palaearctic; Western Palaearctic).

**Asserted distribution in Italy**: North Italy (Scaramozzino 1995); TAA (Cobelli 1899, as *Porizonhostilis* Gravenhorst, 1829); PIE (Frilli and Horstmann 1982, as *Ophionhostilis* (Gravenhorst, 1829)).

#### Barycnemis guttulator

(Thunberg, 1822)

A73B22DA-5A02-5B20-BCC3-E11B381C61F9

##### Distribution

Palaearctic (Eastern Palaearctic; Western Palaearctic).

**Asserted distribution in Italy**: North Italy (Scaramozzino 1995); EMR (Roberti et al. 1965, as *Porizonguttulator* (Thunberg, 1822)).

#### Barycnemis harpura

(Schrank, 1802)

FB45CCF9-D349-5DCA-9928-1041D6F46F7A

##### Distribution

Nearctic; Palaearctic (Eastern Palaearctic; Western Palaearctic).

**Asserted distribution in Italy**: North Italy (Horstmann 1981, Scaramozzino 1995); TAA (Pagliano 2009); PIE (Pagliano 2009); LIG (Pagliano 2009); EMR (Zangheri 1969, as *Leptopygusharpurus* (Schrank, 1802)); SIC (De Stefani 1895, as *Porizonharpurus* (Schrank, 1802)).

#### Barycnemis punctifrons

Horstmann, 1981

E9426D42-5E1F-5CAA-BEBE-38DD19DBB037

##### Distribution

Palaearctic (Eastern Palaearctic; Western Palaearctic).

**Asserted distribution in Italy**: North Italy (Scaramozzino 1995).

#### Diaparsis

Förster, 1869

F67D1C5E-F7DB-5E75-82AA-667E929B0655

#### Diaparsis

Förster, 1869

B42173F1-EF81-556F-8CD2-E22015BE8119

#### Diaparsis (Diaparsis) basalis

Horstmann, 1981

B67BDA07-8985-510B-872C-E3A2D9EDC12A

##### Distribution

Palaearctic (Western Palaearctic).

**Asserted distribution in Italy**: North Italy (Scaramozzino 1995).

#### Diaparsis (Diaparsis) carinifer

(Thomson, 1889)

A6D02954-0696-53E1-AFEA-8FA761BFAE92

##### Distribution

Nearctic; Palaearctic (Eastern Palaearctic; Western Palaearctic).

**Asserted distribution in Italy**: North Italy (Horstmann 1971, Scaramozzino 1995); PIE (Pagliano 2009); EMR (Venturi 1942, as *Thersilochus* [sic] *moderator*); TOS (Venturi 1942, as *Thersilochus* [sic] *moderator*); LAZ (Dysart et al. 1973); SAR (Horstmann 1971, Scaramozzino 1995).

##### Notes

Venturi (1942) reports *Thersilochus* [sic] *moderator* (Linnaeus, 1758) as a parasite of “*Lemamelanopa*” (= *Oulemamelanopus* (Linnaeus, 1758)) (Coleoptera, Chrysomelidae). According to Dysart et al. (1973), whose opinion we follow here, the species reported by Venturi (1942) is *Diaparsiscarinifer* (Thomson, 1889). Venturi (1942) himself noted the oddity of obtaining the parasitoid from a chrysomelid, whereas the records for the species were all from weevils (Curculionidae). It is clear that the author applied an incorrect name (“*moderator*”) to the specimens obtained from *Oulema*, comparing then his results with the host records known for *Porizonmoderator* known at that time. Venturi (1942) does not specify the localities of his records, but his research was carried out in Tuscany and Emilia-Romagna. We assume, therefore, that the parasitoid may be present in these two administrative regions.

#### Diaparsis (Diaparsis) jucunda

(Holmgren, 1860)

CCDEC08F-7188-5527-8400-B8CE51C5857A

##### Materials

- **Type status:** Other material. **Occurrence:** recordedBy: G. G. M. Schulten; individualCount: 1; sex: male; occurrenceID: 504D5A36-5470-5767-8F0E-A3ED4F2D5177; **Location:** country: Italy; countryCode: IT; stateProvince: Latium [LAZ]; municipality: Roma; locality: Albano; **Identification:** identifiedBy: A. Khalaim; **Event:** year: 1986; month: 6; day: 10; verbatimEventDate: 10.vi.1986; **Record Level:** institutionCode: RMNH

##### Distribution

Palaearctic (Eastern Palaearctic; Western Palaearctic).

**Asserted distribution in Italy**: LOM (Haye and Kenis 2004); PIE (Haye and Kenis 2004); LAZ (**new record**).

##### Notes

The specimen from Latium marks the first record for **South Italy**.

#### Diaparsis (Diaparsis) multiplicator

Aubert, 1969

445C26FA-F488-5961-B1E7-81B1CC98060A

##### Materials

- **Type status:** Other material. **Occurrence:** recordedBy: M. Bardiani et al.; individualCount: 4; sex: 3 females, 1 male; occurrenceID: CB937AD1-3F2E-5B6F-A612-5DC7A57658BA; **Location:** country: Italy; countryCode: IT; stateProvince: Lombardy [LOM]; municipality: Mantova; locality: Marmirolo, Bosco della Fontana; verbatimElevation: 25 m; **Identification:** identifiedBy: A. Khalaim; **Event:** samplingProtocol: Malaise trap on canopy (16 m); year: 2008; month: 4-5; day: 29-13; verbatimEventDate: 29.iv–13.v.2008; **Record Level:** institutionCode: FDGC
- **Type status:** Other material. **Occurrence:** recordedBy: G. Lo Giudice; individualCount: 1; sex: female; occurrenceID: E0DAD673-DA41-5773-ABD1-D40C1D1DD608; **Location:** country: Italy; countryCode: IT; stateProvince: Abruzzo [ABR]; municipality: L'Aquila; locality: Santo Stefano; **Identification:** identifiedBy: A. Khalaim; **Event:** year: 2008; month: 5; day: 24; verbatimEventDate: 24.v.2008; **Record Level:** institutionCode: FDGC

##### Distribution

Palaearctic (Eastern Palaearctic; Western Palaearctic).

**Asserted distribution in Italy**: North Italy (Scaramozzino 1995); LOM (**new record**); PIE (Pagliano 2009); South Italy (Scaramozzino 1995); ABR (**new record**); BAS (Pagliano 2009).

#### Diaparsis (Diaparsis) nutritor

(Fabricius, 1804)

647A55CF-11D8-5112-8CFD-7C535929155F

##### Materials

- **Type status:** Other material. **Occurrence:** recordedBy: M. Bardiani; D. Birtele; individualCount: 1; sex: male; occurrenceID: C950398E-2224-5F02-B54F-F2BB453586A4; **Location:** country: Italy; countryCode: IT; stateProvince: Lombardy [LOM]; municipality: Mantova; locality: Marmirolo, Bosco della Fontana; verbatimElevation: 25 m; **Identification:** identifiedBy: A. Khalaim; **Event:** samplingProtocol: Malaise trap (ground); year: 2008; month: 5-6; day: 27-10; verbatimEventDate: 27.v–10.vi.2008; **Record Level:** institutionCode: FDGC
- **Type status:** Other material. **Occurrence:** recordedBy: M. Bardiani; D. Birtele; individualCount: 1; sex: female; occurrenceID: A90D68D4-69DC-53D5-86B4-99A9476DCDE3; **Location:** country: Italy; countryCode: IT; municipality: Mantova; locality: Marmirolo, Bosco della Fontana; verbatimElevation: 25 m; **Event:** samplingProtocol: Malaise trap (ground); year: 2008; month: 6; day: 10-24; verbatimEventDate: 10–24.vi.2008; **Record Level:** institutionCode: FDGC

##### Distribution

Palaearctic (Western Palaearctic).

**Asserted distribution in Italy**: LOM (**new record**); PIE (Frilli and Horstmann 1982, as *Ophionnutritor* Fabricius, 1804).

#### Diaparsis (Diaparsis) rara

Horstmann, 1971

B5980A78-9DCF-5AA1-88C2-B1C1516FCBCE

##### Distribution

Palaearctic (Eastern Palaearctic; Western Palaearctic).

**Asserted distribution in Italy**: North Italy (Scaramozzino 1995, as Diaparsis (Pseudaneuclis) rara Horstmann, 1971).

#### Diaparsis (Diaparsis) temporalis

Horstmann, 1979

BCB2DFAC-7F64-5308-956F-DFE2468EABEA

##### Materials

- **Type status:** Other material. **Occurrence:** recordedBy: D. Inclán; individualCount: 1; sex: female; occurrenceID: D9AD7A44-8705-5C44-A18C-21D840859850; **Location:** country: Italy; countryCode: IT; stateProvince: Tuscany [TOS]; municipality: Siena; locality: Pienza; **Identification:** identifiedBy: A. Khalaim; **Event:** samplingProtocol: Yellow pan trap; year: 2012; month: 4; day: 17-18; verbatimEventDate: 17–18.iv.2012; **Record Level:** institutionCode: FDGC
- **Type status:** Other material. **Occurrence:** recordedBy: F. Di Giovanni leg; individualCount: 2; sex: 1 female, 1 male; occurrenceID: 9A4490A5-0CF8-5C75-8839-E3217C6C3979; **Location:** country: Italy; countryCode: IT; stateProvince: Apulia [PUG]; municipality: Foggia; locality: Emmaus; verbatimCoordinates: 41°28'15" N, 15°27'52"E; verbatimLatitude: 41°28'15" N; verbatimLongitude: 15°27'52"W; decimalLatitude: 41.470833; decimalLongitude: 15.464444; geodeticDatum: WGS84; **Identification:** identifiedBy: A. Khalaim; **Event:** year: 2016; month: 4; day: 30; verbatimEventDate: 30.iv.2016; **Record Level:** institutionCode: FDGC

##### Distribution

Nearctic; Palaearctic (Eastern Palaearctic; Western Palaearctic).

**Asserted distribution in Italy**: North Italy (Scaramozzino 1995); PIE (Pagliano 2009); South Italy (Horstmann 1979, Horstmann 1981, Scaramozzino 1995); TOS (**new record**); PUG (**new record**).

#### Ischnobatis

Förster, 1869

A0F166C6-3AEF-59C1-9A50-717863A829E0

#### Diaparsis (Ischnobatis) stramineipes

(Brischke, 1880)

E330F982-F22D-5B50-B492-EEE0D1034921

##### Distribution

Palaearctic (Eastern Palaearctic; Western Palaearctic).

**Asserted distribution in Italy**: EMR (Zangheri 1969, as *Thersilochus* [sic] *rufiventris* Brischke, 1880).

#### Nanodiaparsis

Horstmann, 1971

8154E31A-AEA9-5BA4-B46E-AB7041E0A873

#### Diaparsis (Nanodiaparsis) aperta

(Thomson, 1889)

ECB55B08-4D0A-5189-9302-3379921804EE

##### Materials

- **Type status:** Other material. **Occurrence:** recordedBy: M. Bardiani; D. Birtele; individualCount: 1; sex: female; occurrenceID: C089C24B-2E05-5288-8DD1-6744EABF730E; **Location:** country: Italy; countryCode: IT; stateProvince: Lombardy [LOM]; municipality: Mantova; locality: Marmirolo, Bosco della Fontana; verbatimElevation: 25 m; **Identification:** identifiedBy: A. Khalaim; **Event:** samplingProtocol: Malaise trap on canopy (17 m); year: 2008; month: 6-7; day: 26-8; verbatimEventDate: 26.vi–8.vii.2008; **Record Level:** institutionCode: FDGC
- **Type status:** Other material. **Occurrence:** recordedBy: F. Di Giovanni; individualCount: 14; sex: 2 females, 12 males; occurrenceID: FE0632FD-6F80-5256-9045-847A6E294DC4; **Location:** country: Italy; countryCode: IT; stateProvince: Apulia [PUG]; municipality: Foggia; locality: Emmaus; verbatimCoordinates: 41°28'15" N, 15°27'52"E; verbatimLatitude: 41°28'15" N; verbatimLongitude: 15°27'52"E; decimalLatitude: 41.470833; decimalLongitude: 15.464444; geodeticDatum: WGS84; **Identification:** identifiedBy: A. Khalaim; **Event:** year: 2016; month: 4; day: 30; verbatimEventDate: 30.iv.2016; habitat: on *Sinapis* sp.; **Record Level:** institutionCode: FDGC

##### Distribution

Palaearctic (Eastern Palaearctic; Western Palaearctic).

**Asserted distribution in Italy**: LOM (**new record**); PUG (**new record**).

##### Notes

**Figure 2.**
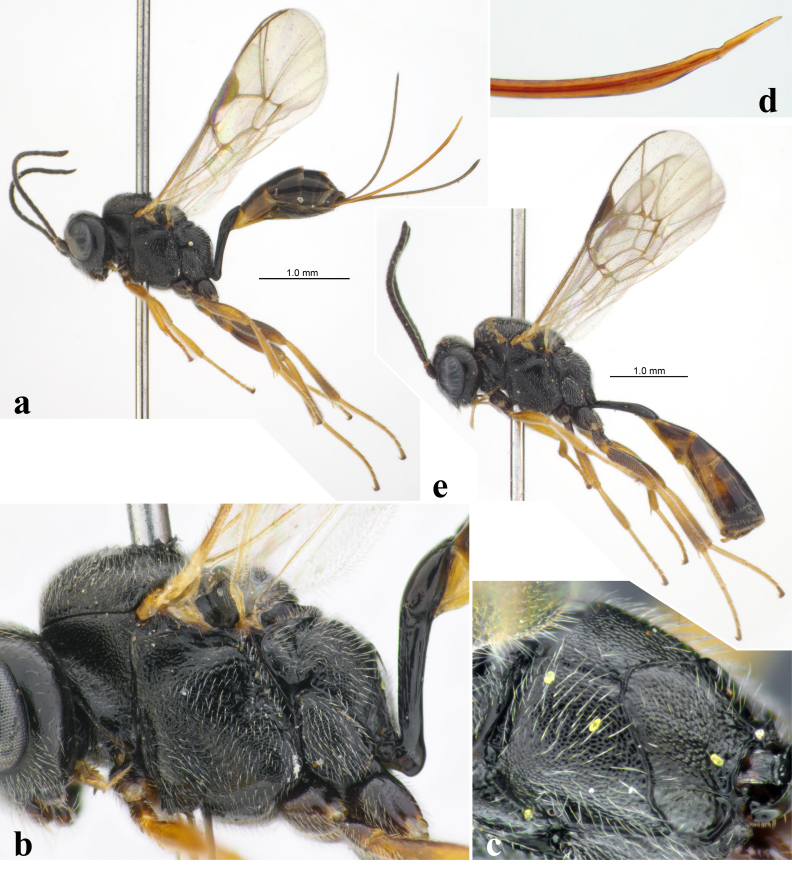
Diaparsis (Nanodiaparsis) aperta (Thomson, 1889); **a-d**: female (Italy); **a** habitus, lateral view; **b** mesosoma and base of metasoma, lateral view; **c** propodeum, dorso-lateral view; **d** apex of ovipositor, lateral view; **e** male (Italy), habitus, lateral view.

The specimens from Lombardy and Apulia mark the first records of this species for **Italy** (Fig. 2).

#### Epistathmus

Förster, 1869

49A7C659-A895-5AF9-9BFD-65B55666F553

#### Epistathmus crassicornis

Horstmann, 1971

6F422400-C20E-5D2F-8C32-65E1246AF40F

##### Materials

- **Type status:** Other material. **Occurrence:** recordedBy: C. van Achterberg; individualCount: 1; sex: male; occurrenceID: 8DC86F7C-8963-5271-AA6D-170EFA726099; **Location:** country: Italy; countryCode: IT; stateProvince: Lombardy [LOM]; municipality: Como; locality: Lanzo d’Intelvi; verbatimElevation: 700 m; **Identification:** identifiedBy: A. Khalaim; **Event:** year: 1982; month: 8; day: 10; verbatimEventDate: 10.viii.1982; habitat: meadow; **Record Level:** institutionCode: RMNH

##### Distribution

Palaearctic (Eastern Palaearctic; Western Palaearctic).

**Asserted distribution in Italy**: North Italy (Scaramozzino 1995); TAA (Horstmann 1971, Khalaim 2016, Khalaim 2019); LOM (**new record**).

#### Gelanes

Horstmann, 1981

AC4E0492-1F4A-5994-BED9-13C161C533E1

#### Gelanes fusculus

(Holmgren, 1860)

64C18DB5-9D8D-5303-999C-1CFEF65A6E69

##### Distribution

Palaearctic (Eastern Palaearctic; Western Palaearctic).

**Asserted distribution in Italy**: TAA (Khalaim and Blank 2011).

#### Heterocola

Förster, 1869

148AEB25-E954-536A-A879-C74D73E2B469

#### Heterocola

Förster, 1869

93CB6963-1B2F-5829-99EE-6FF6F42F8C5B

#### Heterocola (Heterocola) proboscidalis

(Thomson, 1889)

F77CCB2B-980D-5397-9BC5-3862DC895457

##### Distribution

Palaearctic (Eastern Palaearctic; Western Palaearctic).

**Asserted distribution in Italy**: North Italy (Scaramozzino 1995); TAA (Horstmann 1971); EMR (Zangheri 1969).

#### Heterocoloides

Horstmann, 1971

6F09A88C-F7FB-56CB-8E35-C4714642F3A3

#### Heterocola (Heterocoloides) linguaria

(Haliday, 1838)

8AE0EFB2-0C35-53C4-8A98-707FE0F538E0

##### Materials

- **Type status:** Other material. **Occurrence:** recordedBy: M.J. Gijswijt; individualCount: 1; sex: female; occurrenceID: 021AC6F4-63EB-5718-AF3B-173141B73E97; **Location:** country: Italy; countryCode: IT; stateProvince: Abruzzo [ABR]; municipality: L'Aquila; locality: Opi; verbatimElevation: 1100 m; **Identification:** identifiedBy: A. Khalaim; **Event:** year: 1993; month: 6; day: 13; verbatimEventDate: 13.vi.1993; **Record Level:** institutionCode: RMNH

##### Distribution

Palaearctic (Western Palaearctic).

**Asserted distribution in Italy**: North Italy (Horstmann 1971, as Heterocola (Heterocoloides) punctulata (Szépligeti, 1899); Scaramozzino 1995, as Heterocola (Heterocoloides) punctulata (Szépligeti, 1899)); EMR (Zangheri 1969, as *Heterocolapunctulata* (Szépligeti, 1899)); South Italy (Scaramozzino 1995, as Heterocola (Heterocola) linguaris [sic] (Haliday, 1838)); ABR (**new record**).

#### Palpator

Khalaim, 2006

55CF6354-1E03-50FE-9005-90F3EBAFC1E8

#### Palpator sicilicus

Khalaim, 2006

A131BAC1-3E57-5D8C-9273-693C5BCFFD57

##### Distribution

Palaearctic (Western Palaearctic).

**Asserted distribution in Italy**: SIC (Khalaim 2006).

##### Notes

So far, the species is only known from Sicily. The description is in Khalaim (2006).

#### Phradis

Förster, 1869

7DEE6DD6-4BFC-5102-A033-355868D2F9F2

#### Phradis brevicornis

Horstmann, 1971

8107E19D-0584-5EF2-B6FE-178FC7C8B986

##### Distribution

Palaearctic (Eastern Palaearctic; Western Pelearctic).

**Asserted distribution in Italy**: North Italy (Scaramozzino 1995); VEN (Khalaim et al. 2009); TAA (Horstmann 1971, Khalaim et al. 2009).

#### Phradis brevis

(Brischke, 1880)

1C4834DE-64A3-5541-B3FB-D799C5A6C690

##### Distribution

Palaearctic (Eastern Pelearctic; Western Palaearctic).

**Asserted distribution in Italy**: North Italy (Horstmann 1971, Scaramozzino 1995); PIE (Pagliano 2009).

#### Phradis interstitialis

(Thomson, 1889)

BA1C9F5A-D5DC-5DDC-A285-FE8B829CA410

##### Materials

- **Type status:** Other material. **Occurrence:** recordedBy: C. van Achterberg; R. de Vries; individualCount: 1; sex: female; occurrenceID: 0C1F9EE3-E512-5F5D-B2A1-0F0F120E73D5; **Location:** country: Italy; countryCode: IT; stateProvince: Lombardy [LOM]; municipality: Cuneo; locality: Parco Naturale delle Alpi Marittime, Trinità, Ponte del Suffiet; verbatimElevation: 1192 m; verbatimCoordinates: 44°11'427" N, 07°26'264"E; verbatimLatitude: N 44°11'427" N; verbatimLongitude: 07°26'264"E; decimalLatitude: 44.195194; decimalLongitude: 7.457333; geodeticDatum: WGS84; **Identification:** identifiedBy: A. Khalaim; **Event:** samplingProtocol: Malaise trap; year: 2008; month: 6; day: 10-24; verbatimEventDate: 10–24.vi.2008; habitat: near rivulet, edge of wet forest; **Record Level:** institutionID: RMNH

##### Distribution

Palaearctic (Eastern Palaearctic; Western Palaearctic).

**Asserted distribution in Italy**: LOM (Khalaim et al. 2009; **new record**); EMR (Zangheri 1969, as *Isurgusinterstitialis* (Thomson, 1889)); BAS (Khalaim et al. 2009).

#### Phradis minutus

(Bridgman, 1889)

7B7CB5C3-A105-57CC-9EAC-91C87DF990DD

##### Materials

- **Type status:** Other material. **Occurrence:** recordedBy: D. Inclán; individualCount: 1; sex: female; occurrenceID: 201B084D-28B3-52EA-9DDB-089D80E1A3B2; **Location:** country: Italy; countryCode: IT; stateProvince: Tuscany [TOS]; municipality: Siena; locality: San Giovani d’Asso; **Identification:** identifiedBy: A. Khalaim; **Event:** samplingProtocol: Yellow pan trap; year: 2012; month: 4; day: 17-18; verbatimEventDate: 17–18.iv.2012; **Record Level:** institutionCode: FDGC

##### Distribution

Palaearctic (Western Palaearctic).

**Asserted distribution in Italy**: North Italy (Horstmann 1971, Scaramozzino 1995); VEN (Khalaim et al. 2009); TAA (Khalaim et al. 2009); TOS (**new record**).

##### Notes

The specimen from Tuscany marks the first record for **South Italy**.

#### Phradis monticola

Szépligeti, 1899

27B4CCA2-9AAE-5651-9F45-ABD6F3643696

##### Distribution

Palaearctic (Western Palaearctic).

**Asserted distribution in Italy**: North Italy (Scaramozzino 1995); TAA (Horstmann 1971, Khalaim et al. 2009).

#### Phradis morionellus

(Holmgren, 1860)

39C1B9AD-25C4-5C24-B3BE-DF67BE81BB37

##### Materials

- **Type status:** Other material. **Occurrence:** recordedBy: D. Inclán; individualCount: 1; sex: female; occurrenceID: 46E6C618-F875-5C65-98FD-7CF8CA800C6D; **Location:** country: Italy; countryCode: IT; stateProvince: Tuscany [TOS]; municipality: Siena; locality: Asciano; **Identification:** identifiedBy: A. Khalaim; **Event:** samplingProtocol: Yellow pan trap; year: 2012; month: 10; day: 2-5; verbatimEventDate: 02–05.x.2012; **Record Level:** institutionCode: FDGC
- **Type status:** Other material. **Occurrence:** recordedBy: D. Inclán; individualCount: 1; sex: female; occurrenceID: BE1219E3-3181-5D3A-9ADD-34A815252BC2; **Location:** country: Italy; countryCode: IT; stateProvince: Tuscany [TOS]; county: Siena; municipality: Asciano; **Identification:** identifiedBy: A. Khalaim; **Event:** samplingProtocol: Yellow pan trap; year: 2012; month: 3-4; day: 31-1; verbatimEventDate: 31.iii–01.iv.2012; **Record Level:** institutionCode: FDGC
- **Type status:** Other material. **Occurrence:** recordedBy: D. Inclán; individualCount: 1; sex: female; occurrenceID: 79514705-8DCB-504C-A1CD-4AA50380804A; **Location:** country: Italy; countryCode: IT; stateProvince: Tuscany [TOS]; municipality: Siena; locality: Vescona Chiesa; **Identification:** identifiedBy: A. Khalaim; **Event:** samplingProtocol: Yellow pan trap; year: 2012; month: 3-4; day: 31-1; verbatimEventDate: 31.iii–01.iv.2012; **Record Level:** institutionCode: FDGC

##### Distribution

Palaearctic (Eastern Palaearctic; Western Palaearctic).

**Asserted distribution in Italy**: EMR (Zangheri 1969, as *Isurguslanceolatus* Szépligeti, 1899); TOS (**new record**).

##### Notes

The specimens from Tuscany mark the first record for **South Italy**.

#### Phradis nigritulus

(Gravenhorst, 1829)

D6937043-E1E6-5204-B8F3-3E8DC0D46B9A

##### Materials

- **Type status:** Other material. **Occurrence:** recordedBy: C. van Achterberg; R. de Vries; individualCount: 1; sex: female; occurrenceID: 8E0B06E7-53C6-588B-B71C-A25B67150E31; **Location:** country: Italy; countryCode: IT; stateProvince: Piedmont [PIE]; municipality: Cuneo; locality: Parco Naturale delle Alpi Marittime, Valdieri; verbatimElevation: 933 m; verbatimCoordinates: N 44°16'358", E 07°24'048"; verbatimLatitude: 44°16'358" N; verbatimLongitude: 07°24'048" E; decimalLatitude: 44.276611; decimalLongitude: 7.401333; geodeticDatum: WGS84; **Identification:** identifiedBy: A. Khalaim; **Event:** samplingProtocol: Malaise trap; year: 2008; month: 6; day: 10-24; verbatimEventDate: 10–24.vi.2008; habitat: on *Juniperusphoniceae*, on S rock slope; **Record Level:** institutionCode: RMNH
- **Type status:** Other material. **Occurrence:** recordedBy: C. van Achterberg; R. de Vries; individualCount: 2; sex: females; occurrenceID: A40C73A6-0B50-5DD3-9907-5D7CC64F2E90; **Location:** country: Italy; countryCode: IT; stateProvince: Piedmont [PIE]; municipality: Cuneo; locality: Parco Naturale delle Alpi Marittime, Valdieri; verbatimElevation: 933 m; verbatimCoordinates: N 44°16'336", E 07°24'127"; verbatimLatitude: 44°16'336" N; verbatimLongitude: 07°24'127" E; decimalLatitude: 44.276000; decimalLongitude: 7.403528; geodeticDatum: WGS84; **Identification:** identifiedBy: A. Khalaim; **Event:** year: 2008; month: 6; day: 10-24; verbatimEventDate: 10–24.vi.2008; habitat: on *Juniperusphoniceae*, on SE rock slope; **Record Level:** institutionCode: RMNH

##### Distribution

Palaearctic (Eastern Palaearctic; Western Palaearctic).

**Asserted distribution in Italy**: TAA (Khalaim et al. 2009); PIE (**new record**); SIC (Horstmann 1981, Scaramozzino 1995, Khalaim et al. 2009).

#### Phradis punctipleuris

Horstmann, 1971

C197CBA3-E2B4-5A77-BAB0-1DFEFB14FEE2

##### Distribution

Palaearctic (Eastern Palaearctic; Western Palaearctic).

**Asserted distribution in Italy**: North Italy (Scaramozzino 1995); TAA (Khalaim et al. 2009).

#### Phradis thyridialis

Horstmann, 1981

04008A49-2928-5430-B164-4C887C446809

##### Distribution

Palaearctic (Eastern Palaearctic; Western Palaearctic).

**Asserted distribution in Italy**: VEN (Khalaim et al. 2009); LOM (Khalaim et al. 2009).

#### Phradis terebrator

Horstmann, 1981

0ACFFDD8-2EEE-55E7-8D8C-5E03F817FFAB

##### Distribution

Palaearctic (Eastern Palaearctic; Western Palaearctic).

**Asserted distribution in Italy**: North Italy (Scaramozzino 1995).

#### Phradis toreador

Aubert, 1986

8BB4F0DF-D418-5A10-8A57-0EE804567E06

##### Distribution

Palaearctic (Western Palaearctic).

**Asserted distribution in Italy**: Italy (Scaramozzino 1995); PIE (Aubert 1986).

#### Phrudus

Förster, 1869

FDD70506-3306-53E1-8F8D-AC2ECEAD93DD

#### Phrudus badensis

Hilpert, 1987

05E5A314-F2BA-5102-A827-51E0C4C1EE78

##### Distribution

Palaearctic (Eastern Palaearctic; Western Palaearctic).

**Asserted distribution in Italy**: North Italy (Scaramozzino 1995); PIE (Vikberg and Koponen 2000, Pagliano 2009).

#### Phrudus monilicornis

(Bridgman, 1886)

447955D3-E9DD-524B-AD72-EC81259B5B6C

##### Distribution

Indomalayan; Palaearctic (Eastern Palaearctic; Western Palaearctic).

**Asserted distribution in Italy**: VEN (Vikberg and Koponen 2000); TAA (Vikberg and Koponen 2000).

#### Probles

Förster, 1869

34DDD982-D158-5EC5-9139-1D715EB56453

#### Euporizon

Horstmann, 1971

B5F69495-12C3-5E46-951A-14D9319C5F6A

#### Probles (Euporizon) brevicauda

Horstmann, 1981

1BC3613A-28A0-59EA-9C43-CB8344B92428

##### Distribution

Palaearctic (Western Palaearctic).

**Asserted distribution in Italy**: North Italy (Scaramozzino 1995, sub Probles (Microdiaparsis) versutus (Holmgren, 1860)).

##### Notes

The species was listed in Scaramozzino (1995) as a form of *Problesversutus* (Holmgren, 1860). Indeed, Scaramozzino (1995) reports that some of the records of *P.versutus* from northern Italy has to be referred to the *brevicauda* "form" of Horstmann, 1971. See also notes under Probles (Microdiaparsis) versutus (Holmgren, 1860).

#### Probles (Euporizon) exilis

(Holmgren, 1860)

A3C1C045-71AD-56EF-8425-C6D2A39CE645

##### Distribution

Palaearctic (Eastern Palaearctic; Western Palaearctic).

**Asserted distribution in Italy**: North Italy (Scaramozzino 1995); PIE (Pagliano 2009).

#### Probles (Euporizon) extensor

(Aubert, 1971)

03609B52-A7CD-5997-BA2C-345153175163

##### Distribution

Palaearctic (Western Palaearctic).

**Asserted distribution in Italy**: North Italy (Scaramozzino 1995); PIE (Pagliano 2009); LIG (Pagliano 2009).

#### Probles (Euporizon) gilvipes

(Gravenhorst, 1829)

95621004-DD09-5293-ABF7-1F5387C4A57F

##### Distribution

Palaearctic (Western Palaearctic).

**Asserted distribution in Italy**: EMR (Zangheri 1969, as *Diaparsispallipes* (Holmgren, 1860)).

#### Probles (Euporizon) montanus

Horstmann, 1971

4910B4CF-AD95-5EF6-BBCD-28A507E51B9C

##### Distribution

Palaearctic (Western Palaearctic).

**Asserted distribution in Italy**: North Italy (Horstmann 1971, Scaramozzino 1995); PIE (Pagliano 2009); LIG (Pagliano 2009).

#### Probles (Euporizon) nigriventris

Horstmann, 1971

484597FD-D580-5E81-AB28-A1402896CC77

##### Distribution

Palaearctic (Western Palaearctic).

**Asserted distribution in Italy**: North Italy (Horstmann 1971, Scaramozzino 1995); PIE (Pagliano 2009).

#### Probles (Euporizon) rufipes

(Holmgren, 1860)

02A7563F-CA21-5D88-A644-95454DC1BA9E

##### Distribution

Palaearctic (Western Palaearctic).

**Asserted distribution in Italy**: North Italy (Horstmann 1971, Scaramozzino 1995).

#### Probles (Euporizon) tenuicornis

Horstmann, 1981

BB45AB44-F5C0-5D85-9A34-8F223B8EC1AD

##### Distribution

Palaearctic (Western Palaearctic).

**Asserted distribution in Italy**: North Italy (Scaramozzino 1995); PIE (Pagliano 2009).

#### Probles (Euporizon) truncorum

(Holmgren, 1860)

1CF8439E-5915-5B58-B6FC-01864D838D10

##### Distribution

Palaearctic (Western Palaearctic).

**Asserted distribution in Italy**: North Italy (Scaramozzino 1995); TAA (Horstmann 1971).

#### Microdiaparsis

Horstmann, 1971

DF9EBE58-A21F-556F-A86C-1AAB4D6AB127

#### Probles (Microdiaparsis) caudiculatus

Khalaim, 2007

927D0FF3-1327-50E6-8C1E-4AA76A4EAD85

##### Materials

- **Type status:** Other material. **Occurrence:** recordedBy: C. van Achterberg; R. de Vries; occurrenceID: 9964909B-C673-54CA-93AE-9F5E1BB97D2A; **Location:** country: Italy; countryCode: IT; stateProvince: Piedmont [PIE]; municipality: Cuneo; locality: Parco Naturale delle Alpi Marittime, near Palanfré; verbatimElevation: 1488 m; verbatimCoordinates: N 44°11'395", E 07°29'364"; verbatimLatitude: 44°11'395" N; verbatimLongitude: 07°29'364" E; decimalLatitude: 44.194306; decimalLongitude: 7.493444; geodeticDatum: WGS84; **Identification:** identifiedBy: A. Khalaim; **Event:** samplingProtocol: Malaise trap; year: 2008; month: 6; day: 11-2; verbatimEventDate: 11–24.vi.2008; habitat: meadow, edge of forest; **Record Level:** institutionCode: RMNH

##### Distribution

Palaearctic (Eastern Palaearctic; Western Palaearctic).

**Asserted distribution in Italy**: VEN (Khalaim 2007); TAA (Khalaim 2007); PIE (Khalaim 2007; **new record**).

#### Probles (Microdiaparsis) microcephalus

(Gravenhorst, 1829)

7BFEE6F7-5A79-5456-A3DA-7F5B1246FD82

##### Distribution

Palaearctic (Western Palaearctic).

**Asserted distribution in Italy**: North Italy (Horstmann 1971, as *Microdiaparsismicrocephalus* (Gravenhorst, 1829); *Scaramozzino 1995*); PIE (Pagliano 2009); EMR (Zangheri 1969, as *Thersilochus* [sic] *quercetorum* Szépligeti, 1899); SIC (De Stefani 1895, as *Porizonmicrocephalus* Gravenhorst, 1829).

#### Probles (Microdiaparsis) neoversutus

(Horstmann, 1967)

7380B5E3-083F-5C5E-9E06-BC4976546B00

##### Distribution

Palaearctic (Eastern Palaearctic; Western Palaearctic).

**Asserted distribution in Italy**: North Italy (Scaramozzino 1995); PIE (Pagliano 2009).

#### Probles (Microdiaparsis) versutus

(Holmgren, 1860)

5FD798AE-B703-535B-9285-DC1EAAC15EC7

##### Materials

- **Type status:** Other material. **Occurrence:** recordedBy: E. Haeselbarth; individualCount: 1; sex: female; occurrenceID: F473B5D5-6141-5FDD-9E1D-7A75E23A397A; **Location:** country: Italy; countryCode: IT; stateProvince: Trentino-Alto Adige [TAA], South Tyrol; municipality: Bolzano; locality: St. Peter/Ahrntal; verbatimElevation: 1300 m; **Identification:** identifiedBy: A. Khalaim; **Event:** year: 1967; month: 8; day: 2; verbatimEventDate: 25.viii.1967; **Record Level:** institutionCode: ZISP
- **Type status:** Other material. **Occurrence:** recordedBy: L. Colacurcio; individualCount: 1; sex: female; occurrenceID: 8B7704E1-CCA5-59A4-9701-FCBF1CE5A6EB; **Location:** country: Italy; countryCode: IT; stateProvince: Emilia-Romagna [EMR]; municipality: Bologna; locality: Sasso Marconi, Palazzo Rossi; **Identification:** identifiedBy: A. Khalaim; **Event:** year: 2010; month: 2-3; verbatimEventDate: ii–iii.2010; **Record Level:** institutionCode: FDGC
- **Type status:** Other material. **Occurrence:** recordedBy: L. Colacurcio; individualCount: 1; sex: male; occurrenceID: 5BB7D66B-BCB8-509A-BE91-871AB844DA01; **Location:** country: Italy; countryCode: IT; stateProvince: Emilia-Romagna [EMR]; municipality: Bologna; locality: Sasso Marconi, Palazzo Rossi; **Identification:** identifiedBy: A. Khalaim; **Event:** year: 2010; month: 3; verbatimEventDate: 08.iii.2010; **Record Level:** institutionCode: FDGC

##### Distribution

Palaearctic (Eastern Palaearctic; Western Palaearctic).

**Asserted distribution in Italy**: North Italy (Scaramozzino 1995); TAA (Horstmann 1971, as *Microdiaparsisversutus* (Holmgren, 1860); **new record**); PIE (Pagliano 2009); EMR (Zangheri 1969, as *Diaparsisversutus* (Holmgren 1860); **new record**).

##### Notes

The data reported by Scaramozzino (1995) for this species partly include those for Probles (Euporizon) brevicauda Horstmann, 1981, which Scaramozzino (1995) listed as a form of *P.versutus* (Holmgren, 1860).

#### Probles

Förster, 1869

A591FAA5-E0E7-5B22-92D2-2A3573970F9C

#### Probles (Probles) brevivalvis

Horstmann, 1971

152A1521-3655-5C8E-9C52-FE92ECD38228

##### Distribution

Palaearctic (Western Palaearctic).

**Asserted distribution in Italy**: North Italy (Scaramozzino 1995); TAA (Horstmann 1971, Khalaim 2016, Khalaim 2019).

#### Probles (Probles) erythrostomus

(Gravenhorst, 1829)

954118CE-EF2E-5F26-B4CE-584FC0E8AD4B

##### Distribution

Palaearctic (Western Palaearctic).

**Asserted distribution in Italy**: North Italy (Horstmann 1971, Scaramozzino 1995); PIE (Gravenhorst 1829, as *Porizonminator* Gravenhorst, 1829); LIG (Gravenhorst 1829, as *Porizonminator* Gravenhorst, 1829); South Italy (Horstmann 1971, Scaramozzino 1995).

##### Notes

Horstmann (1979) recorded this species from central Italy, which is treated as South Italy according to the division into geographical subregions proposed by Minelli et al. (1993-1995).

#### Probles (Probles) flavipes

(Szépligeti, 1899)

1A3CC9D1-38C4-580E-8546-F66C2C7EC13D

##### Distribution

Palaearctic (Western Palaearctic).

**Asserted distribution in Italy**: North Italy (Scaramozzino 1995); TAA (Horstmann 1971).

#### Rugodiaparsis

Horstmann, 1971

EB06D468-0E18-5EA9-B928-4E3371E939F2

#### Probles (Rugodiaparsis) crassipes

(Thomson, 1889)

87B1E69B-1C05-54AA-9498-005800AB9F57

##### Distribution

Palaearctic (Western Palaearctic).

**Asserted distribution in Italy**: North Italy (Horstmann 1981, Scaramozzino 1995); PIE (Pagliano 2009).

#### Probles (Rugodiaparsis) ruficornis

(Szépligeti, 1899)

65FF0734-DE50-507B-8A95-7297D39813E3

##### Distribution

Palaearctic (Eastern Palaearctic; Western Palaearctic).

**Asserted distribution in Italy**: North Italy (Scaramozzino 1995); PIE (Pagliano 2009).

#### Rhynchoprobles

Horstmann, 1971

A5DA90AE-6F31-58BE-89F4-ACD88E3CBCED

#### Probles (Rhynchoprobles) longisetosus

(Hedwig, 1956)

D62F8AA7-CC9C-5514-B783-F478153234E6

##### Distribution

Palaearctic (Eastern Palaearctic; Western Palaearctic).

**Asserted distribution in Italy**: North Italy (Scaramozzino 1995); TAA (Horstmann 1971).

#### Tersilochus

Holmgren, 1859

04466C0F-78EE-59F7-ADB3-18667213EFB0

#### Gonolochus

Förster, 1869

ACC70B8A-A21C-5001-8903-BFD4E6BA4976

#### Tersilochus (Gonolochus) caudatus

(Holmgren, 1860)

EFB07AE5-598A-5F29-AF03-757FA3FDBB25

##### Materials

- **Type status:** Other material. **Occurrence:** recordedBy: L. Blommers; individualCount: 3; sex: 2 females, 1 male; occurrenceID: 8EEFCB13-1F38-5365-A0DA-96868A98006F; **Location:** country: Italy; countryCode: IT; stateProvince: Trentino-Alto Adige [TAA]; municipality: Trento; locality: San Rocco; **Identification:** identifiedBy: A. Khalaim; **Event:** samplingProtocol: Malaise trap; year: 1994; month: 4-5; day: 21-18; verbatimEventDate: 21.iv–18.v.1994; **Record Level:** institutionCode: RMNH
- **Type status:** Other material. **Occurrence:** recordedBy: C. van Achterberg; R. de Vries; occurrenceID: 0A576653-D328-5660-A56C-8AE61DF8433F; **Location:** country: Italy; countryCode: IT; stateProvince: Piedmont [PIE]; municipality: Cuneo; locality: Parco Naturale delle Alpi Marittime, near Palanfré; verbatimElevation: 1488 m; verbatimCoordinates: N 44°11’395ʹʹ, E 7°29’364ʹʹ; verbatimLatitude: 44°11’395ʹʹ N; verbatimLongitude: 7°29’364ʹʹ E; decimalLatitude: 44.194306; decimalLongitude: 7.493444; geodeticDatum: WGS84; **Identification:** identifiedBy: A. Khalaim; **Event:** samplingProtocol: Malaise trap; year: 2008; month: 6; day: 11-24; verbatimEventDate: 11–24.vi.2008; habitat: meadow, edge of forest; **Record Level:** institutionCode: RMNH
- **Type status:** Other material. **Occurrence:** recordedBy: C. van Achterberg; R. de Vries; individualCount: 1; sex: female; occurrenceID: 63021316-9138-5FD8-A4AA-C6EBA8B462EA; **Location:** country: Italy; countryCode: IT; stateProvince: Piedmont [PIE]; municipality: Cuneo; locality: Parco Naturale delle Alpi Marittime, Trinità, Ponte del Suffiet; verbatimElevation: 1192 m; verbatimCoordinates: N 44°11’427ʹʹ, E 7°26’264ʹʹ; verbatimLatitude: 44°11’427ʹʹ N; verbatimLongitude: 7°26’264ʹʹE; decimalLatitude: 44.195194; decimalLongitude: 7.440667; geodeticDatum: WGS84; **Identification:** identifiedBy: A. Khalaim; **Event:** samplingProtocol: Malaise trap; year: 2008; month: 6; day: 10-24; verbatimEventDate: 10–24.vi.2008; habitat: near rivulet, edge of wet forest; **Record Level:** institutionCode: RMNH
- **Type status:** Other material. **Occurrence:** recordedBy: C. van Achterberg; R. de Vries; individualCount: 1; sex: female; occurrenceID: 3BC544D9-F3A0-580D-A04F-34D2744BC15D; **Location:** country: Italy; countryCode: IT; stateProvince: Piedmont [PIE]; municipality: Cuneo; locality: Parco Naturale delle Alpi Marittime, Trinità, Gias d’Ischietto; verbatimElevation: 1323 m; verbatimCoordinates: N 44°10'317", E 07°27'348"; verbatimLatitude: 44°10'317" N; verbatimLongitude: 07°27'348" E; decimalLatitude: 44.175472; decimalLongitude: 7.459667; geodeticDatum: WGS84; **Identification:** identifiedBy: A. Khalaim; **Event:** samplingProtocol: Malaise trap; year: 2008; month: 6; day: 13-24; verbatimEventDate: 13–24.vi.2008; habitat: near rivulet, ruderal forest meadow; **Record Level:** institutionCode: RMNH

##### Distribution

Palaearctic (Eastern Palaearctic; Western Palaearctic).

**Asserted distribution in Italy**: North Italy (Horstmann 1971, as *Gonolochuscaudatus* (Holmgren, 1860); *Scaramozzino 1995*); TAA (**new record**); PIE (Pagliano 2009, Khalaim 2016, Khalaim 2019; **new record**); EMR (Roberti et al. 1965; Zangheri 1969, as *Thersilocus* [sic] *caudatus* (Holmgren, 1860)).

#### Tersilochus (Gonolochus) rugulosus

Horstmann, 1981

EB1A0362-9453-58F4-9F1F-48074BDDE708

##### Distribution

Palaearctic (Western Palaearctic).

**Asserted distribution in Italy**: South Italy (Scaramozzino 1995); LAZ (Horstmann 1981).

#### Pectinolochus

Aubert, 1960

315C8BC6-6F56-5864-87F1-A3CE6F91D414

#### Tersilochus (Pectinolochus) coeliodicola

Silvestri, 1917

6FEA460B-D56B-5657-BFBD-5A9036402F58

##### Distribution

Palaearctic (Eastern Palaearctic; Western Palaearctic).

**Asserted distribution in Italy**: South Italy (Horstmann 1971, Scaramozzino 1995); CAM (Silvestri 1917, as *Thersilochus* [sic] *coeliodicola* Silvestri, 1917).

#### Tersilochus (Pectinolochus) striola

(Thomson, 1889)

2C1A1D6C-06B5-518C-BAB7-905871C5201A

##### Distribution

Nearctic; Palaearctic (Eastern Palaearctic; Western Palaearctic).

**Asserted distribution in Italy**: North Italy (Scaramozzino 1995); PIE (Pagliano 2009).

#### Tersilochus (Pectinolochus) terebrator

(Horstmann, 1971)

013DBDB7-3897-5A4C-9D71-BE17493FC55D

##### Materials

- **Type status:** Other material. **Occurrence:** recordedBy: C. van Achterberg; R. de Vries; individualCount: 1; sex: female; occurrenceID: E7793537-7F97-5C56-A716-98CDF0A1B1D2; **Location:** country: Italy; countryCode: IT; stateProvince: Piedmont [PIE]; municipality: Cuneo; locality: Parco Naturale delle Alpi Marittime, Trinità, Ponte del Suffiet; verbatimElevation: 1170 m; verbatimCoordinates: N 44°11'553", E 07°26'192"; verbatimLatitude: 44°11'553" N; verbatimLongitude: 07°26'192" E; decimalLatitude: 44.198694; decimalLongitude: 7.438667; geodeticDatum: WGS84; **Identification:** identifiedBy: A. Khalaim; **Event:** samplingProtocol: Malaise trap; year: 2008; month: 6; day: 10-24; verbatimEventDate: 10–24.vi.2008; habitat: near rivulet, edge of wet forest; **Record Level:** institutionCode: RMNH

##### Distribution

Palaearctic (Eastern Palaearctic; Western Palaearctic).

**Asserted distribution in Italy**: PIE (**new record**).

##### Notes

**Figure 3.**
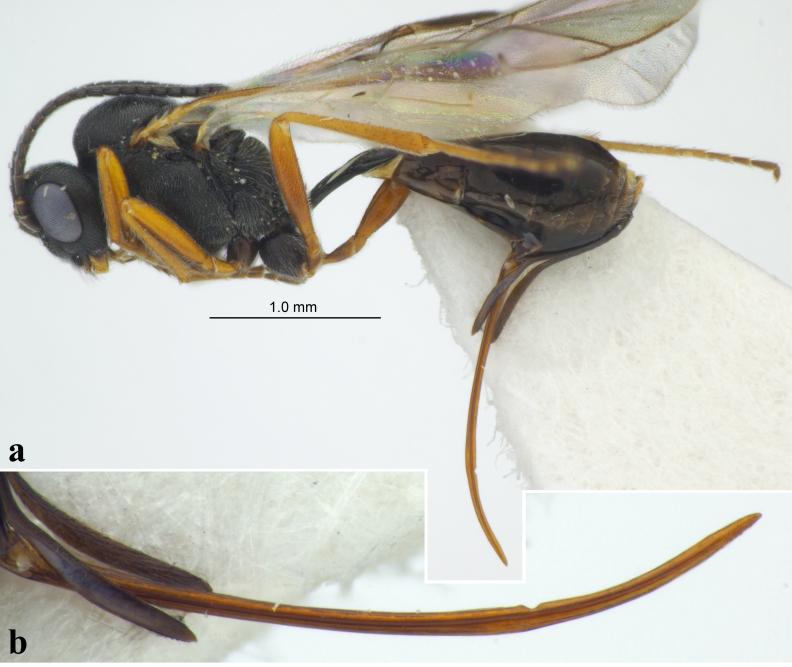
Tersilochus (Pectinolochus) terebrator (Horstmann, 1971), female (Italy); **a** habitus, lateral view; **b** ovipositor, lateral view.

The specimen from Piedmont marks the first record of this species for **Italy** (Fig. 3).

#### Tersilochus

Holmgren, 1859

D8A65393-90AE-5BB6-899C-4BC1AC392805

#### Tersilochus (Tersilochus) brevissimus

Horstmann, 1981

6348E817-EE7B-5680-9090-19AF8AD8E5FD

##### Distribution

Palaearctic (Western Palaearctic).

**Asserted distribution in Italy**: North Italy (Scaramozzino 1995); TAA (Horstmann 1981).

#### Tersilochus (Tersilochus) cognatus

(Holmgren, 1860)

8E929BA0-44D4-5C83-B23D-CD9CB2D87A4F

##### Materials

- **Type status:** Other material. **Occurrence:** recordedBy: L. Blommers; individualCount: 9; sex: 6 females, 3 males; occurrenceID: 54049FC8-2603-5F57-A50E-6F1FADB802F7; **Location:** country: Italy; countryCode: IT; stateProvince: Trentino-Alto Adige [TAA]; municipality: Trento; locality: San Rocco; **Identification:** identifiedBy: A. Khalaim; **Event:** samplingProtocol: Malaise trap; year: 1994; month: 4-5; day: 21-11; verbatimEventDate: 21.iv–11.v.1994; **Record Level:** institutionCode: RMNH
- **Type status:** Other material. **Occurrence:** recordedBy: C. van Achterberg; R. de Vries; individualCount: 1; sex: female; occurrenceID: 088DABF4-18B5-5F67-9B74-1F7BB35EEF1D; **Location:** country: Italy; countryCode: IT; stateProvince: Piedmont [PIE]; municipality: Cuneo; locality: Parco Naturale delle Alpi Marittime, Trinità, Ponte del Suffiet,; verbatimElevation: 1170 m; verbatimCoordinates: N 44°11’553ʹʹ, E 7°26’192ʹʹ; verbatimLatitude: 44°11’553ʹʹ N; verbatimLongitude: 7°26’192ʹʹ E; decimalLatitude: 44.198694; decimalLongitude: 7.438667; geodeticDatum: WGS84; **Identification:** identifiedBy: A. Khalaim; **Event:** samplingProtocol: Malaise trap; year: 2008; month: 6; day: 10-24; verbatimEventDate: 10–24.vi.2008; habitat: near rivulet, edge of wet forest; **Record Level:** institutionCode: RMNH
- **Type status:** Other material. **Occurrence:** recordedBy: C. van Achterberg; R. de Vries; individualCount: 1; sex: female; occurrenceID: 2A72B432-4F86-5943-AFE9-83CCAA259F15; **Location:** country: Italy; countryCode: IT; stateProvince: Piedmont [PIE]; municipality: Cuneo; locality: Parco Naturale delle Alpi Marittime, Trinità, Gias d’Ischietto; verbatimElevation: 1323 m; verbatimCoordinates: N 44°10'317", E 07°27'348"; verbatimLatitude: 44°10'317" N; verbatimLongitude: 07°27'348" E; decimalLatitude: 44.175472; decimalLongitude: 7.459667; geodeticDatum: WGS84; **Identification:** identifiedBy: A. Khalaim; **Event:** samplingProtocol: Malaise trap; year: 2008; month: 6; day: 13-24; verbatimEventDate: 13–24.vi.2008; habitat: near rivulet, ruderal forest meadow; **Record Level:** institutionCode: RMNH

##### Distribution

Palaearctic (Western Palaearctic).

**Asserted distribution in Italy**: North Italy (Horstmann 1981, as *Tersilochusjocator* Holmgren, 1859; Scaramozzino 1995, as *Tersilochusjocator* Holmgren, 1859); TAA (**new record**); PIE (Pagliano 2009, as *Tersilochusjocator* Holmgren, 1859; **new record**); EMR (Zangheri 1969, as *Thersilochus* [sic] *jocator* Holmgren, 1859); South Italy (Scaramozzino 1995, as *Tersilochusjocator* Holmgren, 1859); ABR (Pagliano 2009, as *Tersilochusjocator* Holmgren, 1859).

##### Notes

Holmgren (1859) mentioned the name "*Tersilochus (Porizon) Iocator* Grav." as the type species for the genus *Tersilochus*. However, Gravenhorst (1829) did not describe a new species with this name, but mentioned the *Ichneumonjocator* of Fabricius (1793). As already delineated by Horstmann (1969), *Ichneumonjocator* Fabricius, 1793 is actually an *Eriborus* (Campopleginae) and is currently a synonym of *Eriborusbraccatus* (Gmelin, 1790) (Aubert 1971, Horstmann 2001). As reported by Horstmann (2005), Gravenhorst (1829) misidentified the specimen(s) he included in *Porizonjocator* Fabricius, 1793. Thus, Holmgren, following the interpretation by Gravenhorst (1829) when designating the type species for *Tersilochus*, committed an incorrect subsequent spelling. Those specimens are, in fact, *Tersilochuscognatus* (Holmgren, 1860). Horstmann (1969) wrongly interpreted Holmgren's identification as a deliberate use of an incorrect interpretation (following the 1962 version of the Code). Currently, these cases fall into Article 67.13.1 of ICZN (1999) which clearly states that "*If an author fixes as the type species of a new nominal genus or subgenus a species originally included deliberately in the sense of a misidentification or misapplication by an earlier author of a name which had been previously established [...], the type species fixed by that action is deemed to be a new nominal species [...]; for the name-bearing type of this species see [...*]". With this in mind, what Yu et al. (2016) report is unwarranted, as there is no *jocator* Holmgren, 1859 (unavailable name), because Holmgren (1859) did not deliberately include a misidentification, but simply reported what he thought to be a valid identification by Gravenhorst (1829). It was Horstmann (1969) who deliberately included a wrong identification, creating therefore an unavailable name, *Tersilochusjocator* Horstmann, 1869. This is perfectly explained in Horstmann (2005), which also reported that Holmgren (1859)'s *jocator* should be treated as a misidentification of *cognatus* Holmgren, 1860.

#### Tersilochus (Tersilochus) curvator

Horstmann, 1981

B82E38AE-D984-5B9E-9EC6-AC1814C49476

##### Distribution

Palaearctic (Eastern Palaearctic; Western Palaearctic).

**Asserted distribution in Italy**: North Italy (Scaramozzino 1995, also as Tersilochus (Tersilochus) saltator (Fabricius, 1781)); PIE (Frilli and Horstmann 1982, as *Ophionsaltator* (Fabricius, 1781); Pagliano 2009).

##### Notes

Frilli and Horstmann (1982) and Scaramozzino (1995) report *Tersilochussaltator* (Fabricius, 1781) for Italy. However, *T.saltator* is now considered a species within the genus *Cardiochiles* Nees, 1819 (Braconidae), as *Cardiochilessaltator*. Yu and Horstmann (1997) noticed that specimens of *T.saltator* have been misidentified in the past with *T.curvator*. Therefore, Frilli and Horstmann (1982)'s and Scaramozzino (1995)'s records are, in fact, to be associated with *T.curvator* Horstmann, 1981.

#### Tersilochus (Tersilochus) longicaudatus

Horstmann, 1971

8D97D039-6AB3-50D5-813E-B46CCD35C7F4

##### Materials

- **Type status:** Other material. **Occurrence:** recordedBy: M. Bardiani et al.; individualCount: 4; sex: females; occurrenceID: 054C2A40-F2BB-5148-ACF0-27C1B3305E9D; **Location:** country: Italy; countryCode: IT; stateProvince: Lombardy [LOM]; municipality: Mantova; locality: Marmirolo, Bosco della Fontana; verbatimElevation: 25 m; **Identification:** identifiedBy: A. Khalaim; **Event:** samplingProtocol: Malaise trap on canopy (15–16.5); year: 2008; month: 4; day: 1-15; verbatimEventDate: 01–15.iv.2008; **Record Level:** institutionCode: FDGC

##### Distribution

Palaearctic (Western Palaearctic).

**Asserted distribution in Italy**: North Italy (Scaramozzino 1995); LOM (**new record**); PIE (Pagliano 2009).

#### Tersilochus (Tersilochus) microgaster

(Szépligeti, 1899)

10A449C2-DCE0-5E9D-AEC0-D8D2CD31E518

##### Distribution

Palaearctic (Western Palaearctic).

**Asserted distribution in Italy**: North Italy (Scaramozzino 1995); PIE (Pagliano 2009).

#### Tersilochus (Tersilochus) obscurator

(Aubert, 1959)

18887503-9269-5C69-9FF0-653DA566EAB8

##### Distribution

Palaearctic (Western Palaearctic).

**Asserted distribution in Italy**: North Italy (Scaramozzino 1995).

#### Tersilochus (Tersilochus) ruberi

Horstmann, 1981

9BE03A24-7873-57CC-A963-60B3FDF7A85C

##### Distribution

Palaearctic (Western Palaearctic).

**Asserted distribution in Italy**: South Italy (Scaramozzino 1995); CAM (Horstmann 1981).

#### Tersilochus (Tersilochus) subdepressus

(Thomson, 1889)

3EDDA758-356F-53F7-9268-0B9B98F9A53D

##### Materials

- **Type status:** Other material. **Occurrence:** recordedBy: L. Colacurcio; individualCount: 1; sex: female; occurrenceID: 780B6C24-F671-57AE-9B48-E4166CA8D943; **Location:** country: Italy; countryCode: IT; stateProvince: Emilia-Romagna [EMR]; municipality: Bologna; locality: Casalecchio di Reno, Tizzano; **Identification:** identifiedBy: A. Khalaim; **Event:** year: 2010; month: 3; day: 7; verbatimEventDate: 07.iii.2010; **Record Level:** institutionCode: FDGC
- **Type status:** Other material. **Occurrence:** recordedBy: L. Colacurcio; individualCount: 1; sex: female; occurrenceID: B43E955B-B251-5221-9C15-99A480FC0B7A; **Location:** country: Italy; countryCode: IT; stateProvince: Emilia-Romagna [EMR]; municipality: Bologna; locality: Sasso Marconi, Palazzo Rossi; **Identification:** identifiedBy: A. Khalaim; **Event:** year: 2010; month: 3; day: 8; verbatimEventDate: 08.iii.2010; **Record Level:** institutionCode: FDGC

##### Distribution

Palaearctic (Western Palaearctic).

**Asserted distribution in Italy**: EMR (**new record**).

##### Notes

**Figure 4.**
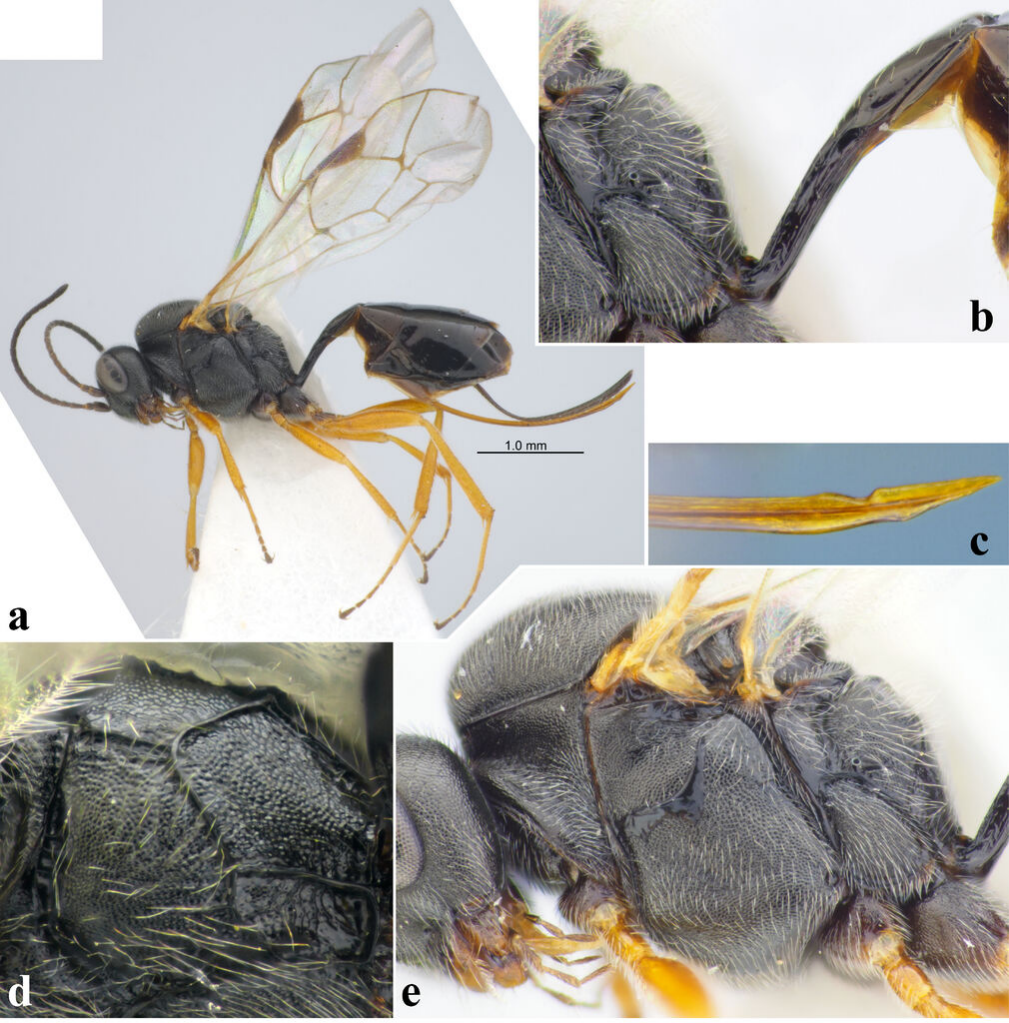
Tersilochus (Tersilochus) subdepressus (Thomson, 1889), female (Italy); **a** habitus, lateral view; **b** posterior part of mesosoma and base of metasoma, lateral view; **c** apex of ovipositor; **d** propodeum, dorso-lateral view; **e** mesosoma, lateral view.

The specimens from Emilia-Romagna mark the first record of this species for **Italy** (Fig. 4).

#### Tersilochus (Tersilochus) triangularis

(Gravenhorst, 1807)

5195F2E4-D795-5EDE-8FB3-24FE7A4A1E0B

##### Distribution

Palaearctic (Western Palaearctic).

**Asserted distribution in Italy**: PIE (Frilli and Horstmann 1982, as *Ophiontriangularis* Gravenhorst, 1807).

#### Tersilochus (Tersilochus) tripartitus

(Brischke, 1880)

1FBCF43A-0BAB-5CF3-A510-8B5D6EB8EFC4

##### Materials

- **Type status:** Other material. **Occurrence:** recordedBy: L. Blommers; individualCount: 1; sex: female; occurrenceID: 439275A0-98EC-5FB2-816B-7CCFC9482B0F; **Location:** country: Italy; countryCode: IT; stateProvince: Trentino-Alto Adige [TAA]; municipality: Trento; locality: San Rocco; **Identification:** identifiedBy: A. Khalaim; **Event:** samplingProtocol: Malaise trap; year: 1994; month: 5; day: 10-11; verbatimEventDate: 10–11.v.1994; **Record Level:** institutionCode: RMNH

##### Distribution

Palaearctic (Western Palaearctic).

**Asserted distribution in Italy**: North Italy (Scaramozzino 1995); TAA (**new record**); PIE (Pagliano 2009).

## Discussion

With the present work, the Italian fauna of Tersilochinae has risen to 77 species, included in 13 genera. This represents a significant increase compared to the 1995 checklist of Italian fauna (Scaramozzino 1995) that listed only 59 species. Despite the increase, 73 out of the 77 species reported in the present work are known from northern Italy, while just 16 species have been recorded for southern regions. Only six species are known from Sicily and only one, *Diaparsiscarinifer* (Thomson, 1889), has been reported for Sardinia so far. No data are yet available for five administrative regions (FVG, MAR, UMB, MOL and CAL). Overall, the current distribution data highlight significant knowledge gaps regarding this subfamily in Italy. It is clear that much of the diversity of the Darwin wasps still remains unexplored, especially in the southernmost part of the Italian peninsula and major islands, where collected data are often scarce and very old. Italy’s situation is particularly challenging due to the complete absence of a centralised national natural history museum (Andreone et al. 2014). Records are scattered amongst museums, universities, public administrations and private foundations, making it extremely difficult to locate identified specimens and, therefore, corroborate previous reported records (Andreone et al. 2014). We stress the importance of checklists as a baseline for future taxonomic, faunistic and ecological studies, directing efforts towards areas that are still little investigated.
